# Tense Bullae and Urticaria in a Woman in Her Sixties

**DOI:** 10.5811/westjem.2014.10.24292

**Published:** 2014-12-02

**Authors:** Katherine C. Wurlitzer, Caleb P. Canders, Angelique S. Campen

**Affiliations:** * Sydney Kimmel Medical College, Philadelphia, Pennsylvania; † UCLA Medical Center, Department of Emergency Medicine, Los Angeles, California

## CASE

**Figure 1:**
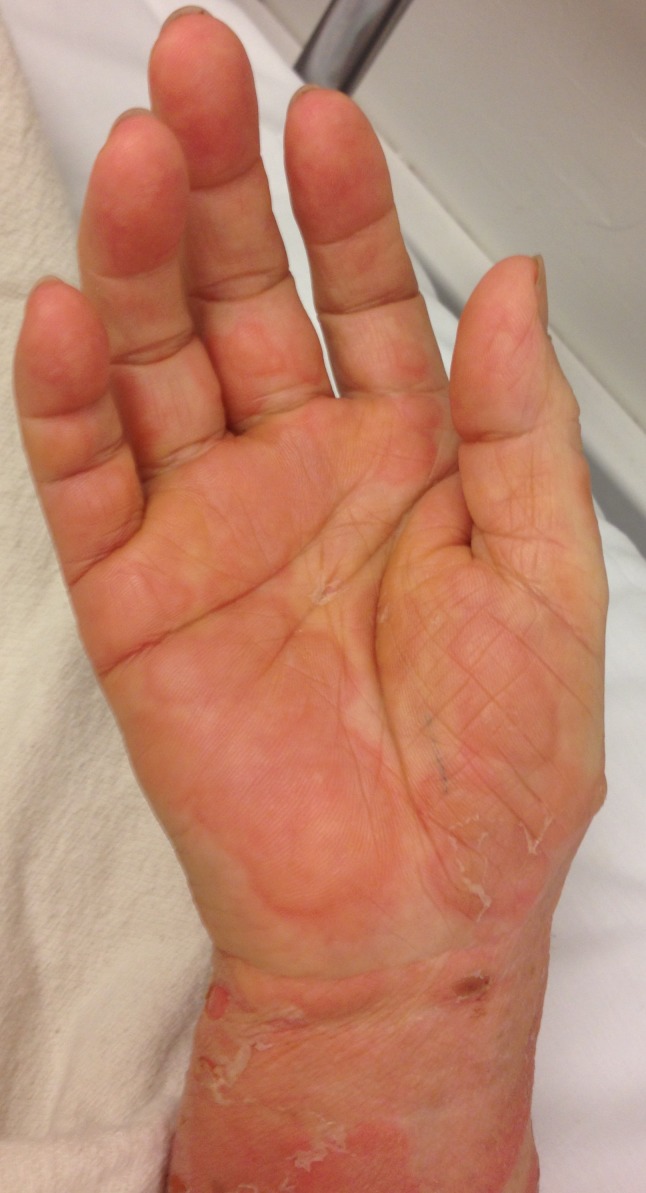
Skin findings include tense bullae on erythematous bases, pruritic plaques on the hands, and urticaria.

**Figure 2:**
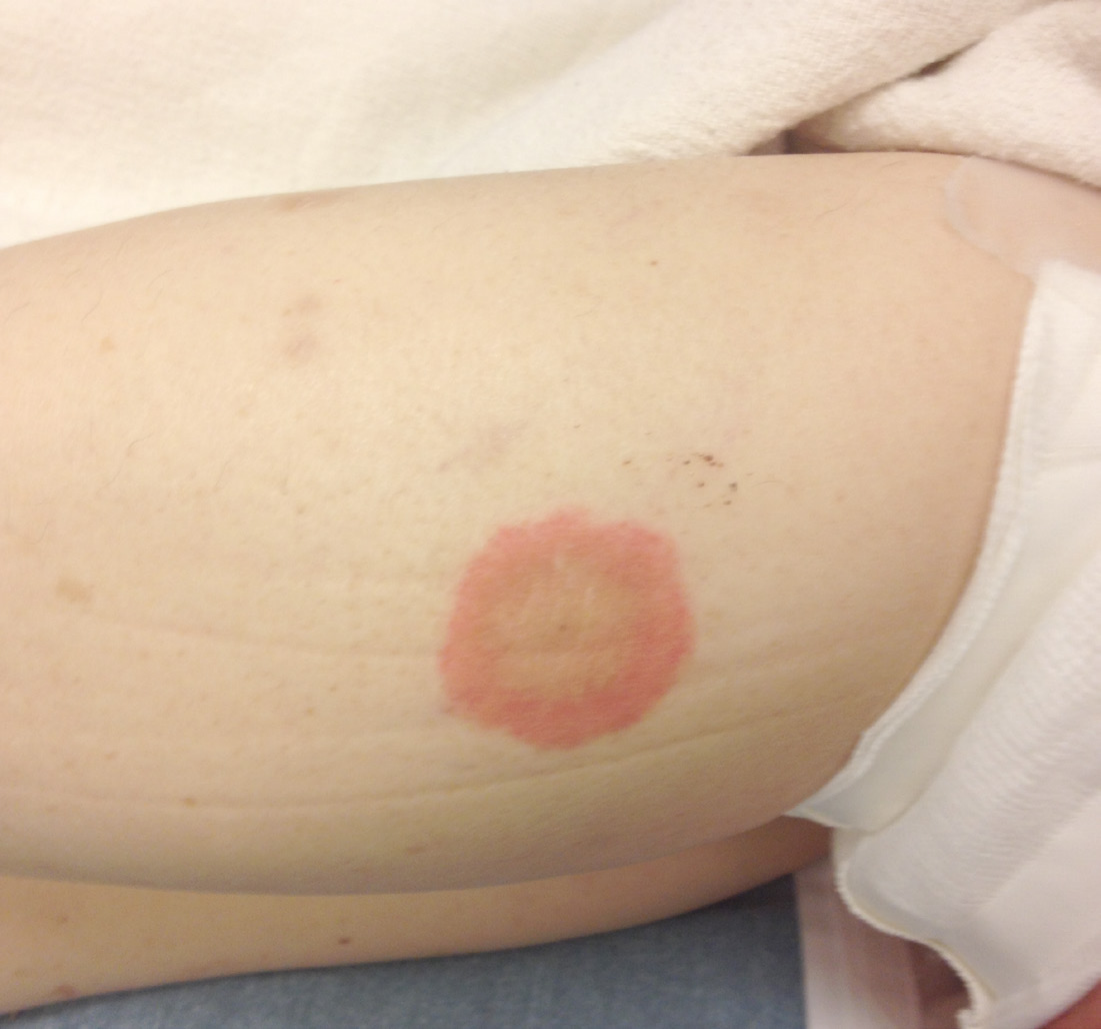
Skin findings include tense bullae on erythematous bases and urticaria on the right inner thigh.

**Figure 3:**
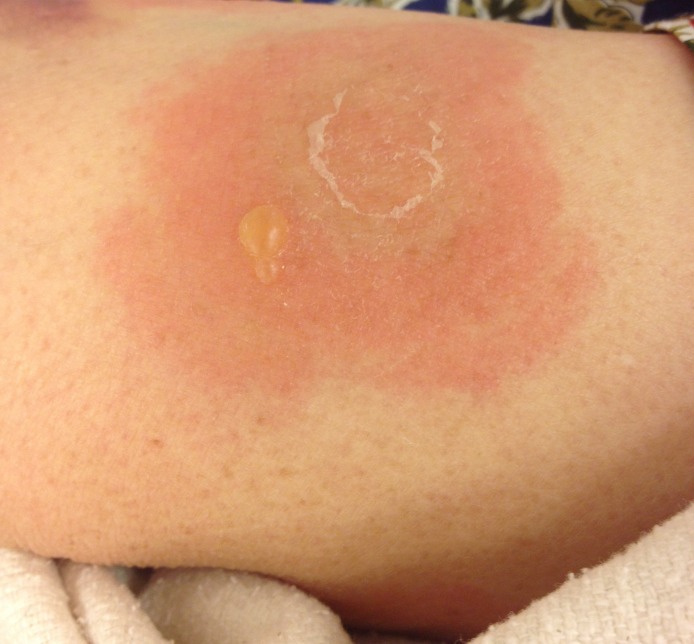
Skin findings include tense bullae on erythematous bases and urticaria on the left anterior thigh.

A 68-year-old woman presented with a pruritic, blistering rash. Three months prior, she had developed itchy lesions on her hands and feet, spreading to her chest and abdomen. For the past week, she had developed painful, tense blisters. The patient had seen her primary care doctor at symptom onset and failed to improve with topical clobetasol proprionate and hydroxyzine. She took amlodipine for blood pressure and denied any new medications or exposures. Physical examination was notable for bullae with mucosal-sparing, urticaria, negative Nikolsky’s sign, and the absence of scarring over ruptured bullae (Figures 1–3). Her complete blood cell count showed an increased number of eosinophils.

## DIAGNOSIS

Bullous pemphigoid, first identified in 1953, is the most common autoimmune blistering disorder.^1^ It has an annual incidence of 6–7 new cases per one million persons, occurs equally in men and women, and typically develops in the seventh or eighth decade of life.^1^–^3^ The disease is characterized by IgG auto-antibodies against the basement membrane hemidesmosome, located at the dermal-epidermal junction.^3^–^4^ Circulating and tissue-bound auto-antibodies bind to target antigens, leading to complement activation, mast cell degranulation, and the release of proteolytic enzymes along the basement membrane, ultimately leading to blister formation.^4^ Risk factors include mechanisms that disrupt the basement membrane, including ultraviolet light, radiation therapy, burns, vaccines, and surgical and accidental traumas.^4^ Certain medications have also been found to induce the disease.^4^ However in 85% patients, no precipitating factor is identified.^4^

In the early, non-bullous phase, patients develop eczematous or urticarial lesions associated with severe pruritus, lasting weeks or months.^1^,^3^–^4^ Patients eventually develop tense blisters that are localized or generalized, and may rupture.^1^,^3^ Only 10–30% of patients have oral involvement.^3^ Proposed diagnostic criteria for bullous pemphigoid include tense blisters or erosions, histologic findings of subepidermal blisters with eosinophil infiltration, and direct immunofluorescence showing linear deposits of IgG and complement along the basement membrane.^1^ Indirect immunofluorescence can also be performed to detect circulating serum auto-antibodies.^3^

First-line treatment consists of topical and systemic corticosteroids and azathioprine. Other treatments include mycophenolate mofetil, leflunomide, cyclophosphamide, methotrexate, dapsone, intravenous immunoglobulin, and plasmapharesis.^5^ Bullous pemphigoid is typically chronic with spontaneous exacerbations and remissions.^5^ It predisposes patients to secondary infections and sepsis, and mortality of bullous pemphigoid ranges 10–40% in the first year following diagnosis.^6^

Our patient was fluid resuscitated and admitted for intravenous steroids. A skin biopsy confirmed the diagnosis. She was eventually discharged home on oral prednisone and mycophenolate mofetil.
